# Supplementary material for: Women’s experiences collecting and accessing water in Guatemala, Honduras, Kenya, and Zimbabwe: A mixed-methods investigation
Source: PLOS Glob Public Health. 2025 Dec 17;5(12):e0004355. doi: 10.1371/journal.pgph.0004355 (PMC12711095; doi:10.1371/journal.pgph.0004355)
Supplement: S1 Checklist — (DOCX) [file pgph.0004355.s001.docx]

|  | **Women’s experiences collecting water in rural Guatemala, Honduras, Kenya, and Zimbabwe:**  **A mixed-methods investigation of water burden**    Bethany A. Caruso, Thea Mink, Madeleine Patrick, Emily Ogutu, Cameron Dawkins, Olivia Bendit, Mahnoor Fatima, Ingrid Lustig, Alicia Macler, Jera White, Alondra Zamora, Jorge Lemus Chávez, Alberto Emanuel Santos López, Héctor Salvador Peña Ramírez, Carlos Daniel Sic, Gladys Ramos, Sandra Antonio, Jazmina Nohemí Irías, Peter Koome, Rohin Otieno Onyango, Petronilla Andiba Otuya, Paul Ruto, Everlyne Atandi, Peter Mwangi, Munyaradzi Damson, Morris Chidavaenzi, Jammaine Jimu, Sithandekile Maphosa, Makaita Maworera, Sheela S. Sinharoy      **S1 Checklist. Standards for Reporting Qualitative Research (SRQR)**  O’Brien BC, Harris IB, Beckman TJ, Reed DA, Cook DA. Standards for reporting qualitative research: a synthesis of recommendations. Academic medicine. 2014 Sep 1;89(9):1245-51. | |
| --- | --- | --- |
|  |  |  |
| **Title and abstract** | | **Page/line no(s).** |
|  | **1. Title** - Concise description of the nature and topic of the study Identifying the study as qualitative or indicating the approach (e.g., ethnography, grounded theory) or data collection methods (e.g., interview, focus group) is recommended | **Title, p.1.** |
|  | **2. Abstract** - Summary of key elements of the study using the abstract format of the intended publication; typically includes background, purpose, methods, results, and conclusions | **Abstract, p.3.** |
|  |  |  |
| **Introduction** | |  |
|  | **3. Problem formulation** - Description and significance of the problem/phenomenon studied; review of relevant theory and empirical work; problem statement | **Intro, p.6.** |
|  | **4. Purpose or research questio**n - Purpose of the study and specific objectives or questions | **Intro, p.6-7.** |
|  |  |  |
| **Methods** | |  |
|  | **5. Qualitative approach and research paradigm** - Qualitative approach (e.g., ethnography, grounded theory, case study, phenomenology, narrative research) and guiding theory if appropriate; identifying the research paradigm (e.g., postpositivist, constructivist/ interpretivist) is also recommended; rationale** | **Methods, p7.**  **[‘Study Design and Motivation’]** |
|  | **6. Researcher characteristics and reflexivity** - Researchers’ characteristics that may influence the research, including personal attributes, qualifications/experience, relationship with participants, assumptions, and/or presuppositions; potential or actual interaction between researchers’ characteristics and the research questions, approach, methods, results, and/or transferability | **Methods, p17-18.**  **[‘Ethics and Reflexivity Statement’]** |
|  | **7. Context** - Setting/site and salient contextual factors; rationale** | **Methods, p8-10. [‘Study Design and Motivation’ & ‘Community and Participant Sample Size and Eligibility, and Setting Specific Details’]** |
|  | **8. Sampling strategy** - How and why research participants, documents, or events were selected; criteria for deciding when no further sampling was necessary (e.g., sampling saturation); rationale** | **Methods, p8-10. [‘Community and Participant Sample Size and Eligibility, and Setting Specific Details’]** |
|  | **9. Ethical issues pertaining to human subjects** - Documentation of approval by an appropriate ethics review board and participant consent, or explanation for lack thereof; other confidentiality and data security issues | **Methods, p17.**  **[‘Ethics and Reflexivity Statement’]** |
|  | **10. Data collection methods** - Types of data collected; details of data collection procedures including (as appropriate) start and stop dates of data collection and analysis, iterative process, triangulation of sources/methods, and modification of procedures in response to evolving study findings; rationale** | **Methods, p10-14.**  **[‘Data Collection Tools and Procedures’,**  **‘Data Collection Training’, & ‘Data Collection and Management’]** |
|  | **11. Data collection instruments and technologies** - Description of instruments (e.g., interview guides, questionnaires) and devices (e.g., audio recorders) used for data collection; if/how the instrument(s) changed over the course of the study | **Methods, p10-12.**  **[‘Data Collection Tools and Procedures’]** |
|  | **12. Units of study** - Number and relevant characteristics of participants, documents, or events included in the study; level of participation (could be reported in results) | **Results, p18.**  **[‘Participant Information’]** |
|  | **13. Data processing** - Methods for processing data prior to and during analysis, including transcription, data entry, data management and security, verification of data integrity, data coding, and anonymization/de-identification of excerpts | **Methods, p13-17.**  **[‘Data Collection and Management’ & ‘Data Analysis’]** |
|  | **14. Data analysis** - Process by which inferences, themes, etc., were identified and developed, including the researchers involved in data analysis; usually references a specific paradigm or approach; rationale** | **Methods, p14-17.**  **[‘Data Analysis’]** |
|  | **15. Techniques to enhance trustworthiness** - Techniques to enhance trustworthiness and credibility of data analysis (e.g., member checking, audit trail, triangulation); rationale** | **Methods, p14-17.**  **[‘Data Analysis’]** |
|  |  |  |
| **Results/findings** | |  |
|  | **16. Synthesis and interpretation** - Main findings (e.g., interpretations, inferences, and themes); might include development of a theory or model, or integration with prior research or theory | **Results, p18-40.**  **[Entire section]** |
|  | **17. Links to empirical data** - Evidence (e.g., quotes, field notes, text excerpts, photographs) to substantiate analytic findings | **Results, p18-40.**  **[Entire section]** |
|  |  |  |
| **Discussion** | |  |
|  | **18. Integration with prior work, implications, transferability, and contribution(s) to the field -** Short summary of main findings; explanation of how findings and conclusions connect to, support, elaborate on, or challenge conclusions of earlier scholarship; discussion of scope of application/generalizability; identification of unique contribution(s) to scholarship in a discipline or field | **Discussion, p41-48.**  **[Entire section]** |
|  | **Limitations** - Trustworthiness and limitations of findings | **Discussion, p46-47.**  **[‘Strengths and Limitations’]** |
|  |  |  |
| **Other** | |  |
|  | **19. Conflicts of interest** - Potential sources of influence or perceived influence on study conduct and conclusions; how these were managed | **Reported in paper metadata.** |
|  | **20. Funding** - Sources of funding and other support; role of funders in data collection, interpretation, and reporting | **Reported in paper metadata.** |
|  |  |  |
|  |  |  |
|  |  |  |
