# Supplementary material for: Women’s experiences collecting and accessing water in Guatemala, Honduras, Kenya, and Zimbabwe: A mixed-methods investigation
Source: PLOS Glob Public Health. 2025 Dec 17;5(12):e0004355. doi: 10.1371/journal.pgph.0004355 (PMC12711095; doi:10.1371/journal.pgph.0004355)
Supplement: S2 Table — (DOCX) [file pgph.0004355.s004.docx]

**Women’s experiences collecting water in rural Guatemala, Honduras, Kenya, and Zimbabwe:**

**A mixed-methods investigation of water burden**

Bethany A. Caruso, Thea Mink, Madeleine Patrick, Emily Ogutu, Cameron Dawkins, Olivia Bendit, Mahnoor Fatima, Ingrid Lustig, Alicia Macler, Jera White, Alondra Zamora, Jorge Lemus Chávez, Alberto Emanuel Santos López, Héctor Salvador Peña Ramírez, Carlos Daniel Sic, Gladys Ramos, Sandra Antonio, Jazmina Nohemí Irías, Peter Koome, Rohin Otieno Onyango, Petronilla Andiba Otuya, Paul Ruto, Everlyne Atandi, Peter Mwangi, Munyaradzi Damson, Morris Chidavaenzi, Jammaine Jimu, Sithandekile Maphosa, Makaita Maworera, Sheela S. Sinharoy

**S2 Table**. Water Journey estimated time comparison and discordance, by country (n=36)

| **S2 Table (part a).** Water Journey estimated time comparison and discordance, by country (n=36) | | | | | | | | | | | | | |
| --- | --- | --- | --- | --- | --- | --- | --- | --- | --- | --- | --- | --- | --- |
|  | **Guatemala** | | | | | |  | **Honduras** | | | | | |
|  | Any | | Drinking | | Other uses | |  | Any | | Drinking | | Other uses | |
|  | n | % | n | % | n | % |  | n | % | n | % | n | % |
| **Total Comparisons^1^** | 4 | | 3 | | 1 |  |  | 5 |  | 3 |  | 2 |  |
| **Time** |  |  |  |  |  |  |  |  |  |  |  |  |  |
| ***Underestimates*** | **1** | **25.0** | **0** | **0.0** | **1** | **100.0** | | **0** | **0.0** | **0** | **0.0** | **0** | **0.0** |
| ≥60 minute underestimate | 1 | 25.0 | 0 | 0.0 | 1 | 100.0 | | 0 | 0.0 | 0 | 0.0 | 0 | 0.0 |
| 30-59 minute underestimate | 0 | 0.0 | 0 | 0.0 | 0 | 0.0 | | 0 | 0.0 | 0 | 0.0 | 0 | 0.0 |
| 16-29 minute underestimate | 0 | 0.0 | 0 | 0.0 | 0 | 0.0 | | 0 | 0.0 | 0 | 0.0 | 0 | 0.0 |
| ***Estimate +/- 15 minutes of actual time*** | **3** | **75.0** | **3** | **100.0** | **0** | **0.0** | | **5** | **100.0** | **3** | **100.0** | **2** | **100.0** |
| ≤15 minute underestimate | 3 | 75.0 | 3 | 100.0 | 0 | 0.0 | | 3 | 60.0 | 3 | 100.0 | 0 | 0.0 |
| ≤15 minute overestimate | 0 | 0.0 | 0 | 0.0 | 0 | 0.0 | | 2 | 40.0 | 0 | 0.0 | 2 | 100.0 |
| ***Overestimates*** | **0** | **0** | **0** | **0.0** | **0** | **0.0** | | **0** | **0.0** | **0** | **0.0** | **0** | **0.0** |
| ≥60 minute overestimate | 0 | 0.0 | 0 | 0.0 | 0 | 0.0 | | 0 | 0.0 | 0 | 0.0 | 0 | 0.0 |
| 30-59 minute overestimate | 0 | 0.0 | 0 | 0.0 | 0 | 0.0 | | 0 | 0.0 | 0 | 0.0 | 0 | 0.0 |
| 16-29 minute overestimate | 0 | 0.0 | 0 | 0.0 | 0 | 0.0 | | 0 | 0.0 | 0 | 0.0 | 0 | 0.0 |
| **Discordance** |  | |  | |  | |  | |  | |  | |  |
| ***No time discordance*** | **1** | **25.0** | **1** | **33.3** | **0** | **0.0** | | **1** | **20.0** | **1** | **33.3** | **0** | **0.0** |
| Estimated and actual both ≤ 30min | 1 | 25.0 | 1 | 33.3 | 0 | 0.0 | | 1 | 20.0 | 1 | 33.3 | 0 | 0.0 |
| Estimated and actual both > 30min | 0 | 0.0 | 0 | 0.0 | 0 | 0.0 | | 0 | 0.0 | 0 | 0.0 | 0 | 0.0 |
| ***Time discordance*** | **3** | **75.0** | **2** | **66.7** | **1** | **100.0** | | **4** | **80.0** | **2** | **66.7** | **2** | **100.0** |
| Estimate at or under 30min, actual over 30min | 2 | 50.0 | 2 | 66.7 | 0 | 0.0 | | 2 | 40.0 | 2 | 66.7 | 0 | 0.0 |
| Estimate over 30min, actual at or under 30min | 1 | 25.0 | 0 | 0.0 | 1 | 100.0 | | 2 | 40.0 | 0 | 0.0 | 2 | 100.0 |
| ^1^ Data from 10 women were excluded because they did not collect water to bring home (9 Guatemala, 1 Kenya) and from 10 women because they did not provide estimated times (5 Guatemala, 1 Honduras, 4 Zimbabwe). Data from 38 women were excluded because their water source type was not the same for their estimated and measured water journey times (5 Guatemala, 10 Honduras, 9 Kenya, 14 Zimbabwe). For the Guatemala participant who completed two water journeys, only her water journey to collect water was included. | | | | | | | | | | | | | |

| **Table S2 (part b).** Water journey estimated time comparison and discordance, by country (n=36) | | | | | | | | | | | | | | | | | | | | |
| --- | --- | --- | --- | --- | --- | --- | --- | --- | --- | --- | --- | --- | --- | --- | --- | --- | --- | --- | --- | --- |
|  | **Kenya** | | | | | |  | **Zimbabwe** | | | | | |  | **Total** | | | | | |
|  | Any | | Drinking | | Other uses | |  | Any | | Drinking | | Other uses | |  | Any | | Drinking | | Other uses | |
|  | n | % | n | % | n | % |  | n | % | n | % | n | % |  | n | % | n | % | n | % |
| **Total Comparisons^1^** | 12 |  | 12 |  | 0 |  |  | 15 | | 12 |  | 3 |  |  | 36 |  | 30 |  | 6 |  |
| **Time** |  |  |  |  |  |  |  |  |  |  |  |  |  |  |  |  |  |  |  |  |
| ***Underestimates*** | **2** | **16.7** | **2** | **16.7** | **0** | **0.0** | | **1** | **6.7** | **1** | **8.3** | **0** | **0.0** | | **4** | **11.1** | **3** | **10.0** | **1** | **16.7** |
| ≥60 minute underestimate | 2 | 16.7 | 2 | 16.7 | 0 | 0.0 | | 0 | 0.0 | 0 | 0.0 | 0 | 0.0 | | 3 | 8.3 | 2 | 6.7 | 1 | 16.7 |
| 30-59 minute underestimate | 0 | 0.0 | 0 | 0.0 | 0 | 0.0 | | 0 | 0.0 | 0 | 0.0 | 0 | 0.0 | | 0 | 0.0 | 0 | 0.0 | 0 | 0.0 |
| 16-29 minute underestimate | 0 | 0.0 | 0 | 0.0 | 0 | 0.0 | | 1 | 6.7 | 1 | 8.3 | 0 | 0.0 | | 1 | 2.8 | 1 | 3.3 | 0 | 0.0 |
| ***Estimate +/- 15 minutes of actual time*** | **1** | **8.3** | **1** | **8.3** | **0** | **0.0** | | **8** | **53.3** | **5** | **41.7** | **3** | **100.0** | | **17** | **47.2** | **12** | **40.0** | **5** | **83.3** |
| ≤15 minute underestimate | 1 | 8.3 | 1 | 8.3 | 0 | 0.0 | | 5 | 33.3 | 2 | 16.7 | 3 | 100.0 | | 12 | 33.3 | 9 | 30.0 | 3 | 50.0 |
| ≤15 minute overestimate | 0 | 0.0 | 0 | 0.0 | 0 | 0.0 | | 3 | 20.0 | 3 | 25.0 | 0 | 0.0 | | 5 | 13.9 | 3 | 10.0 | 2 | 33.3 |
| ***Overestimates*** | **9** | **75** | **9** | **75** | **0** | **0.0** | | **6** | **40.0** | **6** | **50.0** | **0** | **0.0** | | **15** | **41.7** | **15** | **50.0** | **0** | **0.0** |
| ≥60 minute overestimate | 7 | 58.3 | 7 | 58.3 | 0 | 0.0 | | 1 | 6.7 | 1 | 8.3 | 0 | 0.0 | | 8 | 22.2 | 8 | 26.7 | 0 | 0.0 |
| 30-59 minute overestimate | 2 | 16.7 | 2 | 16.7 | 0 | 0.0 | | 1 | 6.7 | 1 | 8.3 | 0 | 0.0 | | 3 | 8.3 | 3 | 10.0 | 0 | 0.0 |
| 16-29 minute overestimate | 0 | 0.0 | 0 | 0.0 | 0 | 0.0 | | 4 | 26.7 | 4 | 33.3 | 0 | 0.0 | | 4 | 11.1 | 4 | 13.3 | 0 | 0.0 |
| **Discordance** |  | |  | |  | |  | |  | |  | |  | |  | |  | |  | |
| ***No time discordance*** | **11** | **91.7** | **11** | **91.7** | **0** | **0.0** | | **11** | **73.3** | **11** | **91.7** | **0** | **0.0** | | **24** | **66.7** | **24** | **80.0** | **0** | **0.0** |
| Estimated and actual both ≤ 30min | 0 | 0.0 | 0 | 0.0 | 0 | 0.0 | | 1 | 6.7 | 1 | 8.3 | 0 | 0.0 | | 3 | 8.3 | 3 | 10.0 | 0 | 0.0 |
| Estimated and actual both > 30min | 11 | 91.7 | 11 | 91.7 | 0 | 0.0 | | 10 | 66.7 | 10 | 83.3 | 0 | 0.0 | | 21 | 58.3 | 21 | 70.0 | 0 | 0.0 |
| ***Time discordance*** | **1** | **8.3** | **1** | **8.3** | **0** | **0.0** | | **4** | **26.7** | **1** | **8.3** | **3** | **100.0** | | **12** | **33.33** | **6** | **20.0** | **6** | **100.0** |
| Estimate at or under 30min, actual over 30min | 0 | 0.0 | 0 | 0.0 | 0 | 0.0 | | 1 | 6.7 | 1 | 8.3 | 0 | 0.0 | | 5 | 13.9 | 5 | 16.7 | 0 | 0.0 |
| Estimate over 30min, actual at or under 30min | 1 | 8.3 | 1 | 8.3 | 0 | 0.0 | | 3 | 20.0 | 0 | 0.0 | 3 | 100.0 | | 7 | 19.4 | 1 | 3.3 | 6 | 100.0 |
| ^1^ Data from 10 women were excluded because they did not collect water to bring home (9 Guatemala, 1 Kenya) and from 10 women because they did not provide estimated times (5 Guatemala, 1 Honduras, 4 Zimbabwe). Data from 38 women were excluded because their water source type was not the same for their estimated and measured water journey times (5 Guatemala, 10 Honduras, 9 Kenya, 14 Zimbabwe). For the Guatemala participant who completed two water journeys, only her water journey to collect water was included. | | | | | | | | | | | | | | | | | | | | |
