# Supplementary material for: Women’s experiences collecting and accessing water in Guatemala, Honduras, Kenya, and Zimbabwe: A mixed-methods investigation
Source: PLOS Glob Public Health. 2025 Dec 17;5(12):e0004355. doi: 10.1371/journal.pgph.0004355 (PMC12711095; doi:10.1371/journal.pgph.0004355)
Supplement: Attachment — Submitted filename: Caruso WJ Review Comment Responses PLoS GPH-2025 30MAY25.docx [file pgph.0004355.s007.docx]

To Whom in May Concern,

RE: " Women’s experiences collecting and accessing water in Guatemala, Honduras, Kenya, and Zimbabwe: A mixed-methods investigation " (PGPH-D-25-00317)

First, we are grateful to the reviewers for the time spent reviewing this manuscript.

Second, please find our point-by-point responses to all comments below, which proved helpful in improving the quality of this manuscript.

We have uploaded both ‘clean’ and ‘tracked changes’ versions of the manuscript to enable easier re-review by all. We hope these revisions are acceptable. Please do not hesitate to contact me with any questions.

We are grateful for your consideration and look forward to hearing from you.

On behalf of all authors,

Bethany Caruso

**Responses to Comments**

| **Journal Requirements** | | |
| --- | --- | --- |
| **Comments / requested revisions** | **Authors’ response** | **Additions /edits to manuscript (as applicable)** |
| J1.  Please include a complete copy of PLOS’ questionnaire on inclusivity in global research in your revised manuscript. Our policy for research in this area aims to improve transparency in the reporting of research performed outside of researchers’ own country or community. The policy applies to researchers who have travelled to a different country to conduct research, research with Indigenous populations or their lands, and research on cultural artefacts. The questionnaire can also be requested at the journal’s discretion for any other submissions, even if these conditions are not met.  Please find more information on the policy and a link to download a blank copy of the questionnaire here: <https://journals.plos.org/globalpublichealth/s/best-practices-in-research-reporting>. Please upload a completed version of your questionnaire as Supporting Information when you resubmit your manuscript. | J1.  We have re-uploaded a copy of the PLOS’ questionnaire on inclusivity in global research as requested.  Note that it was already submitted in the initial submission as S1 Checklist. | J1.  NA |

| **Reviewer 1 Comments** | | |
| --- | --- | --- |
| **Comments / requested revisions** | **Authors’ response** | **Additions /edits to manuscript (as applicable)** |
| **R1.1**  Congratulations on an excellent piece of work. The work is very well written, well researched, and well presented. The findings re-reminded me why I began my own journey within the Gender-WASH space.  I have only a few minor comments to assist in the clarity of several points within the manuscript. | **R1.1**  We are very appreciative of the reviewer and their time spent reading and providing comments on this paper. The paper is stronger as a result.  We have provided responses to each of the comments below in turn. | **R1.1**  NA |
| **R1.2**  Results Table 2: It might be valuable to label the community numbers with a letter and number to avoid confusion (e.g H1 for Honduras community 1) | **R1.2**  We appreciate this feedback to improve clarity. We have made the recommended adjustments to the labels in Table 2. | **R1.2**  See Tables 2a-2d |
| **R1.3**  - Results Figure 2: I think the colours for the R-graphs might be attached to countries. If so, could this be added to the caption? | **R1.3**  Yes, the colors are linked to specific countries! The omission of a legend was an oversight. We have added a legend to label the colors and countries. | **R1.3**  See Figure 2. |
| **R1.4**  - Discussion: Is there anything more about age or life stage that could be included within the discussion? | **R1.4**  We appreciate this suggestion. OUr small, convenience sample limits our own ability to do further analyses, however we agree age and life stage are worth noting. We have expanded the limitations section in the discussion to frame our limitations and note future work that could be done. | **R1.4**  See ‘Strengths and ‘Limitations’ section in discussion. The following was added:  ‘The small convenience samples limit our ability to conduct analysis by age or life course strata. For example, while the greatest proportion of participants in Guatemala and Honduras were unmarried with partners and most participants in Kenya and Zimbabwe were married, we are not able to confirm if the water collection responsibilities and experiences we observed are most typical for women of these life stage groups from these countries compared to women of any other life course stages. Further, we did not collect data with any women under age 18 and therefore are missing their experiences. Future work could explore if and how water collection roles, responsibilities, and burdens vary by age and/or life course stages within countries. Such insights could reveal if water fetching burdens are for all women to bear, or if specific groups of women are more likely to shoulder it.” |
| **R1.5**  - Discussion: You mention some challenges with JMP indicators related to multiple sources for multiple uses, is it worth mentioning the challenges about estimating distance using time? Or do you think that the difficulties in Kenya with animals not going along for the research-water collection negate this? | **R1.5**  We have expanded the discussion to advise caution for those who may want to estimate distance using time data. | **R1.5**  The below was added to the discussion section:  “Further, because we learned about various time-consuming activities taking place at or on the way to the water sources—like watering animals, digging in river beds to extract water, and laundering clothing—we also suggest caution if assuming that distance to a source can be estimated based on either measured or self-reported time. Our data show that the time involved in water collection can include more than just the journey to and from the source, and that the journey times can vary based on the environments.” |
| **R1.6**  - Discussion: Is there an opportunity to connect the final paragraph in the discussion back to gender-transformative WASH practices? | **R1.6**  We now have discussed gender-transformative wash practices in the conclusion paragraph as recommended. | **R1.6**  See conclusion paragraph in discussion (underlined is new text):  “While water is necessary to sustain life and thus should be considered a source of nourishment, we show how a lack of adequate and accessible water was actually depleting, draining women of energy and time, and posing risks to their well-being. Crider and Ray (2022) argue that research in the sector often focuses on women for their instrumental value, or how women can support programming, instead of focusing on women’s intrinsic worth(9). Relatedly, MacArthur and colleagues (2023) offer a framework showing that water, sanitation, and hygiene (WASH) programs can either operate with such instrumental objectives or—alternatively—can have gender-transformative objectives, defined as those that “actively seek to transform the gender norms, structures, and dynamics which perpetuate inequalities within households, communities, and institutions” (71). We agree that programing that seeks to prevent the perpetuation of inequalities is important. We also acknowledge that such approaches may not be possible for those implementing WASH programs to deliver or achieve. As such, we amplify an additional point by MacArthur and colleagues, who note that a more practicable approach may be to both understand existing gender norms and ensure that programs ‘do-no-harm’ by not leveraging or perpetuating these norms for programmatic gains. Through our use of quantitative and qualitative research methods, we centered women’s lived experiences, which have intrinsic value, in hopes of understanding the types of harms to women that could be averted and the potential benefits that women could gain if water programing was more gender aware. The results of our study reinforce the need to redouble efforts to improve water access in low-resource settings, focusing such improvement efforts on providing gender-specific benefits, eliminating harm (even if unintended), and rigorously assessing the impacts of such efforts on women’s lives.” |
| **R1.7**  - Limitations: You may want to consider a short note about the sampling with regards to drawing generalizable conclusions from these selected communities | **R1.7**  We are in agreement. Our second sentence in the section already indicates this point.  We also think that the addition made in regard to age and life stage (as per the reviewer’s suggestion) underscore some of the limitations to generalizability. | **R1.7**  Slight change made for clarity; See ‘Strengths and ‘Limitations’ section in discussion (underlined is new text):  “Our mixed-methods study provides a comprehensive understanding of women’s experiences accessing water sources using novel and state-of-the-art approaches, though limitations exist. Community sampling was purposive, and individual sampling was by convenience, thus data may not be representative, and are not generalizable ~~ility may be limited.~~’ |
| **R1.8**  - References: Check references for consistency in approach. | **R1.8**  We have checked references as advised; all are Vancouver. | **R1.8**  NA |
