# Supplementary material for: Women’s experiences collecting and accessing water in Guatemala, Honduras, Kenya, and Zimbabwe: A mixed-methods investigation
Source: PLOS Glob Public Health. 2025 Dec 17;5(12):e0004355. doi: 10.1371/journal.pgph.0004355 (PMC12711095; doi:10.1371/journal.pgph.0004355)
Supplement: Attachment — Submitted filename: Caruso WJ Review Comment Responses PLoS GPH-2025 3OCT25.docx [file pgph.0004355.s008.docx]

To Whom in May Concern,

RE: " Women’s experiences collecting and accessing water in Guatemala, Honduras, Kenya, and Zimbabwe: A mixed-methods investigation " (PGPH-D-25-00317)

We are grateful to the reviewer for the time spent reviewing this manuscript.

Our point-by-point responses to all comments below, which proved helpful in improving the quality of this manuscript.

We have uploaded both ‘clean’ and ‘tracked changes’ versions of the manuscript to enable easier re-review by all. We hope these revisions are acceptable. Please do not hesitate to contact me with any questions.

We are grateful for your consideration and look forward to hearing from you.

On behalf of all authors,

Bethany Caruso

**Responses to Comments**

| **Journal Requirements** | | |
| --- | --- | --- |
| **Comments / requested revisions** | **Authors’ response** | **Additions /edits to manuscript (as applicable)** |
| J1.  If the reviewer comments include a recommendation to cite specific previously published works, please review and evaluate these publications to determine whether they are relevant and should be cited. There is no requirement to cite these works unless the editor has indicated otherwise. | J1.  Many thanks for this comment and clarification. The reviewer recommended papers. We have integrated and cited those that are relevant, which we believe strengthens some of our points. | J1.  NA |
| J2  Please review your reference list to ensure that it is complete and correct. If you have cited papers that have been retracted, please include the rationale for doing so in the manuscript text, or remove these references and replace them with relevant current references. Any changes to the reference list should be mentioned in the rebuttal letter that accompanies your revised manuscript. If you need to cite a retracted article, indicate the article’s retracted status in the References list and also include a citation and full reference for the retraction notice. | J2.  We have reviewed the reference list.  No papers have been removed.  Two papers have been added that we were not aware of, but that were shared by the reviewer. | J2.  NA |

| **Reviewer 2 Comments** | | |
| --- | --- | --- |
| **Comments / requested revisions** | **Authors’ response** | **Additions /edits to manuscript (as applicable)** |
| **Overall.**  This is a valuable and well-written paper that makes an important contribution to understanding women’s experiences with water collection across diverse contexts. The study design is innovative, and the findings are relevant for both research and practice. Below are suggested revisions to better contextualize the findings and communicate their importance. | Thank you for your time reviewing. The comments are appreciated and the clarity we are encouraged to provide certainly strengthens the paper, from the methods through the results and discussion. | **NA** |
| **R2.1**  Throughout: Ensure usage of “data” as plural (e.g., at line 102, it should read “data are,” not “data is”). | **R2.1**  Good catch. We re-checked the manuscript and changed this and a few other incidences where we mistakenly treated data as singular. | **R2.1**  A handful of edits were made throughout to verbs related to ‘data’ |
| **R2.2**   Abstract: Consider removing the reference to understanding the “gendered burden of this labor.” While the study compellingly documents substantial water-related burdens among women, there is no comparative gender analysis. This does not lessen the contribution of the paper; reframing the aim will better align expectations with the study’s findings. | **R2.2**  Noted. We have made edits as requested. | **R2.2**  **Underlined text indicates addition/edit:**  “A current and comprehensive investigation of women’s water collection experiences is needed to understand *their* burden of this labor.” |
| **R2.3**  Introduction, line 104: This section could be strengthened by noting that GPS-based approaches to estimating collection time and distance may also have limitations, since the Euclidean distance between a household and its primary water source often does not reflect the route individuals take. See: <https://ij-healthgeographics.biomedcentral.com/articles/10.1186/s12942-016-0062-8>. | **R2.3**  This is a useful paper we had not seen. We have added a sentence to discuss Euclidean distance. We have also cited the paper as further evidence of a points we had made elsewhere in the introduction. | **R2.3**  **Underlined text indicates addition/edit:**  ‘Similarly, GIS methods to determine Euclidean distance—from homes to the water source—have been found to underestimate the distances traveled compared to GPS methods that can measure the actual routes taken.’ |
| **R2.4**  Introduction, lines 109–110: Consider strengthening the motivation for this study by emphasizing how improved data on water collection can inform policy and practice, rather than framing the opportunity to address a data gap as inherently meaningful. | **R2.4**  We have edited the noted sentence based on the reviewer suggestion. | **R2.4**  **Underlined text indicates addition/edit:**  “Improving understanding of the burden of water collection—specifically the energy and time required and distance traveled—could inform policy, practice and investment related to water infrastructure, including decisions about whether to install piped water systems or where best to position community-level sources.’ |
| **R2.5**Methods: Specify the type of mixed-methods design. The description suggests a convergent parallel design. | **R2.5**  Indeed it is--We specified in the methods and also added a citation. | **R2.5**  **Underlined text indicates addition/edit:**  ‘This convergent parallel mixed methods study(49) used (a) go-along, in-depth interviews (IDIs), (b) semi-structured observation, (c) activity-tracking smart watches, and (d) scales to elucidate women’s experiences going to water sources (Figure 1).’ |
| **R2.6**  Methods: Ensure reporting aligns with COREQ (<https://onlinelibrary.wiley.com/pb-assets/assets/17416612/COREQ_Checklist-1556513515737.pdf>) and SRQR (<https://journals.lww.com/academicmedicine/fulltext/2014/09000/Standards_for_Reporting_Qualitative_Research__A.21.aspx>) standards. Key missing details include number of coders, procedures for ensuring coding reliability, and reflexivity. | **R2.6**  We have added the SRQR checklist. While both SPQR and COREQ are strong checklists, we have elected to use SPQR here. It is more current and incorporated COREQ when it was developed, along with independent expert review. We have also added a reflexivity statement to align with SRQR. | **R2.6**  **Underlined text indicates addition/edit:**  ‘Results are reported according to the Standards for Reporting Qualitative Research (50) (SRQR; See **S1 Checklist**).’ |
| **R2.7**  Methods: Explain the rationale for selecting a multi-site design (e.g., to capture diverse geographic, climatic, and socioeconomic contexts) and whether variation in experiences across sites was expected. | **R2.7**  We have further expanded on the rationale provided in the first section of the methods section. Specifically, we had previously indicated that the work took place in four countries where programming took place. We further indicated that we agreed it would be useful to carry out research in all four areas to understand how experiences varied and to best inform the programing in each area. Further, we added that we expected that women’s experiences would be different in each country based on what was known about the geography, season, and water access in each area. | **R2.7**  **Underlined text indicates addition/edit:**  ‘Research took place across four countries (Guatemala, Honduras, Kenya, and Zimbabwe) in communities where World Vision is delivering its Strong Women, Strong World: Beyond Access (SWSW) programming. The SWSW program seeks to increase women’s empowerment and improve women's well-being through an integrated intervention that includes water improvements and economic empowerment training and support. The SWSW program theorizes that by providing better water services, women will have increased access to water, saving them time and energy and enabling them to use water, time, and energy for other purposes, including economic activities. World Vision hypothesized that women in the SWSW program areas expend substantial time and energy to retrieve limited quantities of water. To test this hypothesis, Emory University worked with World Vision USA and local World Vision offices in Guatemala, Honduras, Kenya, and Zimbabwe to design the present study; Emory and the World Vision partners collectively agreed that research would be useful in all four countries where the program takes place to discern if and how experiences differed across these settings and to best inform the ongoing programs. We expected experiences to vary across countries based on known differences in geography, season, and water access. To carry out this research, each World Vision country office identified a local learning partner to contribute research expertise and lead data collection activities: Centro Universitario de Occidente de la Universidad de San Carlos de Guatemala, the National Autonomous University of Honduras, St. Paul’s University in Kenya, and Datalyst Africa in Zimbabwe.’ |
| **R2.8**  Methods: In Kenya, refer to the language as Swahili rather than Kiswahili – the current phrasing would be like saying interviews were conducted in Español rather than Spanish. Review language naming in other contexts to ensure appropriateness. | **R2.8**  Changes made. | **R2.8** |
| **R2.9**  Methods: State whether data collection coincided with rainy or dry seasons. The timing of interviews could strongly influence experiences and should be acknowledged. | **R2.9**  We have now indicated the season of data collection the methods for each country. | **R2.9**  **Underlined text indicates addition/edit:**  ‘Research in Guatemala took place in seven rural communities in the departments of Quetzaltenango and Totonicapán, mountainous regions in the southern part of the country during the rainy season.’  ‘Research in Honduras took place in six communities across two areas—one rural and one peri-urban—in the department of El Paraíso in eastern Honduras during the rainy season.’  ‘Research in Kenya took place in six rural communities across Isiolo and Samburu Counties during the dry season.’  ‘Research in Zimbabwe took place in six rural communities in Gwanda and Nyanga districts. The districts are drought-prone, and data were collected during the dry season.’ [No new text- season had already been noted for Zimbabwe]. |
| **R2.10**  Methods:  a.) Indicate what women were asked to do during the go-along interviews (e.g., to walk to their usual water source and collect water as they typically would).  b.) Describe information interviewers were trained to elicit and suggested discussion prompts. | **R2.10**  a.) We have added more information regarding what women were asked to do during the go-along interviews.  b.) We have added information to describe the information interviewers were trained to elicit. | **R2.10**  **Underlined text indicates addition/edit:**  “In our study, go-along interviews started in participant’s homes, continued while going to and from the water source (during pauses in walking or at the source), and concluded back at participant’s homes. Participants were asked to go to the water source they would normally be going to on that day and to carry out the activities they would typically carry out. The interview started while participants were at their homes to build rapport before the journey and to not slow the participant or contribute to their exhaustion while walking. Example topics included those that pertained to participants’ typical water-collection related routines, like where and when participants typically collect water, if they go in groups or alone, what activities they carry out either at the source or en route, what challenges they have come across, and what they do with children while collecting. During the journey, the data collectors were guided to make specific observations and, as needed, ask clarifying questions based on observations, including if what they were seeing was typical. For example, data collectors were asked to observe and describe water containers used during the journey, the terrain traversed, the process of water collection, activities carried out in addition to water collection (e.g., breaks, feeding children), encounters with animals or other people, and if the participant experienced any injuries.” |
| **R2.11**  Methods, Section 2.3: Consider including the full interview guides and prompts as supplementary material. | **R2.11**  All tools for the study are already included as a supplement (See S1 Tools). | **R2.11**  **NA** |
| **R2.12**  Methods, line 241: Provide more detail on piloting. Were tools piloted with enumerators or participants? What modifications were made in response to the piloting exercise? | **R2.12**  We have added information on piloting as suggested. | **R2.12**  **Underlined text indicates addition/edit:**  ‘In the last days of training, tools and procedures were piloted prior to data collection to ensure clarity, contextual appropriateness, and their usability. In Guatemala, Honduras, Kenya, and Zimbabwe, initial piloting was conducted among the enumerator teams to identify potential challenges in administering the tools. In Kenya and Zimbabwe, additional pilot testing was carried out with community members from areas adjacent to the main study sites. This process helped the teams identify questions that needed re-wording to strengthen clarity, required additional probing, or were poorly translated. Piloting with the community members also helped the teams in Kenya and Zimbabwe to estimate the duration of each data collection activity. Based on the pilot learning, the teams revised the tools and adjusted the data collection schedule accordingly. Data generated during the pilot phase was excluded from the final analysis.’ |
| **R2.13**  Methods, lines 273–274: Include the full list of key themes used in the analysis. Clarify how themes were selected, whether they were updated iteratively as transcripts were read, and how coding was conducted. | **R2.13**  We have elaborated on the rapid thematic analysis as suggested. | **R2.13**  **Underlined text indicates addition/edit:**  ‘To learn about women’s practices, experiences, and perceptions of water collection, and the environments and factors that influence them, (aim 1; activities ‘a’ and ‘b’ in Figure 1), we conducted a rapid thematic analysis of all transcripts from go-along interviews and observation notes. The themes were chosen a priori based on the prompts in the data collection tools. Themes selected included: timing of trips to water sources, number of trips to water sources, methods used for water carriage, terrain encountered, perceived risks and experienced harms due to water collection/visiting water sources, the accompaniment of children when going to water sources and why (e.g., need to care for children, children assisted), additional work carried out at or on the way to or from water sources, perceived quality of water and any steps taken to treat water. Team member and co-author MP used Excel to organize data by creating four country-specific matrices that present themes by community. MP read each transcript and all observation notes, and any information relevant to a theme was summarized in the appropriate theme cell for community.  After data summarization by community for each country was complete, the matrices were carefully reviewed. MP and BC discussed the matrices and made iterative updates in three cases to combine themes that were closely related for clarity. Specifically, the themes “Number of trips” and “timing of trips” were combined because the timing of trips was dependent in part on the number of trips. “Terrain” and “risk” were combined because the risks identified by participants were closely related to the terrain and read as redundant when separated. “Perceived water quality” and “water treatment” were combined because participants typically spoke of the two themes together and because we had limited data on water treatment. After each country matrix was adjusted, MP created summaries for each theme by country. These summaries were then used to create one table with a country profile for each theme.’ |
| **R2.14**  Methods:  a.) Clarify who read the transcripts and created the community profiles.  b.) What does it mean that transcripts were “organized by the community”? Provide detail on how profiles were constructed, and when and how community meetings were hosted. | **R2.14**  a.) We have added text to clarify who read the transcripts and created the community profiles.  b.) The phrase “organized by the community” was a typo. It should have read “organized by community” (meaning that we looked at data at the community level first, before doing so at the country level). Our edits to this section have fixed this typo; the sentence is now removed. | **R2.14**  **Underlined text indicates addition/edit:**  ' Team member and co-author MP used Excel to organize data by creating four country-specific matrices that present themes by community. MP read each transcript and all observation notes, and any information relevant to a theme was summarized in the appropriate theme cell for community.  After data summarization by community for each country was complete, the matrices were carefully reviewed.’ |
| **R2.15**  a.) Methods, line 296: Clarify whether estimated journey time was recorded before or after water collection.  b.) Reflect on how the data collection process itself may have influenced the results. For example, did participants ever choose a nearer water source to reduce the burden on enumerators, potentially leading to shorter travel times? Conversely, could talking with interviewers during the journey have slowed walking pace and resulted in longer recorded times? | **R2.15**  a.) Estimated journey time was asked before the water collection journey. This has been clarified in the methods.  b.) We have reflected on how the data collection process itself may influenced results, though this reflection is in the limitations section. | **R2.15**  a.)  **Underlined text indicates addition/edit:**  [Methods] “To determine actual water collection time, distance, caloric expenditure, total elevation ascent, and water volume and weight carried for the entire water journey (both to and from water sources)…”  [Limitations] “While those who participated in the study were asked to carry out their water journey as they normally would, we know that our presence may have influenced the source they chose to visit and what they did along the way. For example, some women in Kenya decided to go to unimproved sources despite having a new community-level bore-hole and others mentioned often taking animals with them but none did for the journeys that were observed and recorded. It is unclear if routines were changed because of participation in data collection, but it is possible. Thus, our data may include journeys that are different than what they would have been had we not been there, and could be either longer or shorter. It is also possible that the participants' speed was different because we were with them. Specifically, they could have moved more slowly because they were answering questions or adjusting to the pace of data collection team members who were not accustomed to the journey. Or, they could have moved faster, feeling pressure to complete the trip or deciding to not linger in conversation with others, bathe, or carry out other activities along the way.” |
| **R2.16**  Results, line 335: Indicate whether classification of improved/unimproved sources was based on JMP criteria or participants’ self-reports. | **R2.16**  The classification was by our research team; we see how this could be perceived otherwise and have edited accordingly. | **R2.16**  **Underlined text indicates addition/edit:**  “…and almost half (42; 46%) indicated that they collected drinking water from a source that we classified as unimproved (as per JMP definition(60)).” |
| **R2.17**  Results, lines 346–347: Clarify whether values represent percentage differences or percentage-point differences. | **R2.17**  We have edited for clarity as suggested. | **R2.17**  **Underlined text indicates addition/edit:**  ‘The proportions of women using surface water for ‘other uses’ were greater than the proportions using it for drinking in both Guatemala (+21 percentage points) and Zimbabwe (+23 percentage points).’ |
| **R2.18**  a.) Results, line 354: Specify during which season(s) data were collected.  b.) Clarify whether seasonal variation was probed by interviewers or arose spontaneously. | **R2.18**  a.) We have specified the seasons when data were collected in the methods (in response to point (R2.9).  b.) Seasonal variation was not specifically probed by interviewers and arose spontaneously. Interviewers asked if there were any water sources used apart from the water source being visited on the day participants were being followed. Seasonal variation usually arose in response to this question, as the water sources could become usable or non-usable depending on the season. Additionally, interviewers asked how many times per week a water source was visited, which sometimes led participants to indicate that season influenced the frequency of their water collection.  We have added clarification that season was not probed. | **R2.18**  **Underlined text indicates addition/edit:**  ‘Research in Guatemala took place in seven rural communities in the departments of Quetzaltenango and Totonicapán, mountainous regions in the southern part of the country during the rainy season.’  ‘Research in Honduras took place in six communities across two areas—one rural and one peri-urban—in the department of El Paraíso in eastern Honduras during the rainy season.’  ‘Research in Kenya took place in six rural communities across Isiolo and Samburu Counties during the dry season.’  ‘Research in Zimbabwe took place in six rural communities in Gwanda and Nyanga districts. The districts are drought-prone, and data were collected during the dry season.’ [No new text- season had already been noted for Zimbabwe].  ‘Participants also described other sources they would frequent depending on need or season (discussion of season was spontaneous, not probed).’ |
| **R2.19**  Results, lines 340–342: Information on number of trips per day appears later in the manuscript. Consider consolidating these sections for thematic consistency. | **R2.19**  We have elected to keep the data in lines 340-342 where it is as it is quantitative and is providing insights related to questions in the demographic survey. That said, your point is noted and we have added a note that indicates that the frequency of water collection as described by the participants is elsewhere. | **R2.19**  **Underlined text indicates addition/edit:**  ‘On average, women reported collecting water almost daily (5.6 days/week); though the mean number of reported collection days was lower in Honduras compared to the other countries (Guatemala 6.4 days; Honduras 4.1 days; Kenya 5.7 days; Zimbabwe 5.9 days) (See Table 2b and section ‘Frequency of trips’ for further elaboration from participants about how frequently they collect water and why).’ |
| **R2.20**  Results, line 431: State whether distances refer to round-trip or one-way journeys. | **R2.20**  These were round trip. We have clarified in the text. | **R2.20**  **Underlined text indicates addition/edit:**  “The mean round-trip distance for all water journeys was 3.5km.” |
| **R2.21**  Results, line 451: Clarify whether terrain challenges were described by participants, observed by enumerators, or interpreted by analysts. This level of transparency should be applied to all descriptive findings. | **R2.21**  All descriptive findings came directly from the observation data or were described by the participants, which is now better clarified in the methods section based on a pervious comment by this reviewer. No interpretation was done, other than sorting the data into the appropriate categories and summarizing what was in the data.  The results paragraphs are broad overviews of the theme, though we have aimed to be clear on what was observed and what was stated. These paragraphs summarize Tables 2a,b,c, and d, where we were more specific about delineating what was described vs observed. Where participants described risks that were not observed, it is noted that this was reported by participants (e.g., Guatemala: “In 2 communities (G2, G6), women stated that in the rain, rivers would overflow and become dangerous.” Zimbabwe, “Participants in 2 communities (Z4, Z5) said motorbikes go by quickly making it dangerous.”) | **R2.21**  **NA** |
| **R2.22**  Discussion, line 583: Consider citing an additional relevant exception (see: <https://www.journals.uchicago.edu/doi/abs/10.1086/696531>). | **R2.22**  This is a valuable paper that we had missed. We have added as noted.  We have also found it useful to expand on other aspects of the discussion. | **R2.22**  **Underlined text indicates addition/edit:**  ‘Participants in previous research typically traveled less than 1km with measured times of less than one hour(33, 35, 41-43) (exceptions: 2018 study in Benin (62); 2014 study in Mozambique)(45); previous data tracking caloric expenditure is dated(7) and elevation ascent data could not be found.’  ‘Alternatively, even if closer sources are made available, women may just frequent them more often or shoulder larger loads of water (62). They may get more water but the reductions in time and energy may not be enough to enable engagement in other activities (e.g., economic)(62). Or, women may still use farther or unimproved sources if a newer improved one is not always available (as observed in our study among Kenyan women), requires payment (as noted in Benin(62)), or does not meet their specific preferences or needs (as noted in India(18), Ghana(75), and observed in our study among women from Guatemala who carried heavy clothing loads to and from the river for washing despite access to other sources).’ |
| **R2.23**  Discussion, lines 577–585: Instead of only emphasizing the novelty of elevation ascent data, discuss its practical significance. What do these findings imply for policy and practice? Should elevation be measured in future studies? | **R2.23**  We have expanded this section to note implications for policy and practice. | **R2.23**  **Underlined text indicates addition/edit:**  ‘This study not only provides data that is novel and presents an approach for others to collect similar data in the future, but it also illuminates and expands on our understanding of the burden shouldered for water collection and carrying out work that requires water access. Even if not feasible to mimic the methods of this study in other locations or at scale, those drafting policies and delivering water services can still glean importance insights to inform their work. Specifically, even simple estimates of time, distance, energy, and elevation burden should be considered when assessing the appropriateness of what water services can and should be delivered and who should be prioritized in gaining access to such services.’ |
