# Supplementary material for: Women’s experiences collecting and accessing water in Guatemala, Honduras, Kenya, and Zimbabwe: A mixed-methods investigation
Source: PLOS Glob Public Health. 2025 Dec 17;5(12):e0004355. doi: 10.1371/journal.pgph.0004355 (PMC12711095; doi:10.1371/journal.pgph.0004355)
Supplement: Attachment — Submitted filename: Review Comment_PLOS_PGPH-D-25-00317_29.10.2025.docx [file pgph.0004355.s009.docx]

**Review Comment**

**Paper Title:** Women’s experiences collecting and accessing water in Guatemala, Honduras, Kenya, and Zimbabwe: A mixed-methods investigation

**Paper ID:** PGPH-D-25-00317

**General Remarks**

This manuscript presents a mixed-methods study examining women's water collection experiences in four low-resource countries, combining qualitative insights (go-along interviews and observations) with quantitative measures (smart watches and scales) to quantify time, distance, energy, and other burdens. The research provides real-time evidence, addressing an important gap in understanding the gendered impacts of water insecurity, with implications for policy and interventions. The title is well-worded to capture the central theme of the paper. The abstract is concise and informative, the introduction provides a solid literature review, and the methods section details unique approaches using technology for precise measurements.

The strengths of the article include the multi-country scope, integration of mixed methods, and focus on underrepresented aspects like cognitive burden and energy expenditure – providing research-driven evidence to overlooked but important nuances. The collaboration with local partners also enhances contextual relevance. Other observations and corrections are highlighted below under respective subsections:

**Abstract**

Line 41: "Mean caloric expenditure was 231cal (range: 36 (Guatemala) – 952 (Zimbabwe))." Units could be clarified for scientific accuracy. "Cal" is used, but in nutrition/energy contexts, it's typically "kcal" (kilocalories) to avoid confusion with small calories. Smartwatch data may likely estimate kcal. Kindly re-check.

Line 30: Replace “experiences is” with “experiences are”

Lines 38–42: The ranges (e.g., for time: 13 min in Guatemala to 287 min in Kenya) imply overall minimum-maximum values for specific countries. Clarify if these are extrema across all participants or per-country mins/maxes.

**Keywords**: There are no keywords. Include about 4 – 6 keywords, arranged in alphabetical order

**Introduction**

Line 57: Change ‘responsibility’ to ‘responsibilities’

Line 67: Change ‘injury’ to ‘injuries’

Line 85: Add ‘a’ before ‘…more precise’

Line 94: Write ‘roundtrip’ to ‘round-trip’

Line 102 – 103: Write ‘Traveled’ as ‘Travelled’

**Methods**

Line 134: Figure 1 is missing

Line 150: Change ‘sample of individuals who were eligible’ to ‘sample of eligible individuals’

Line 187: Change ‘was’ to ‘were’

Line 208: Address missing Figure 1

Other specific remarks:

1. In Methods (Line 155; Page 8, Line 155): "In Guatemala, 95% of the population (91% rural; 98% urban) has at least basic drinking water service (i.e., from an improved source requiring no more than 30 minutes roundtrip for collection) (4)." You may revise as "*In Guatemala, 95% of the population (91% rural; 98% urban) has at least basic drinking water service, defined as access to an improved source requiring no more than 30 minutes round-trip for collection per WHO/UNICEF JMP* (4)."
2. The use of convenience sampling [Line 150] and purposive community selection by World Vision [Line 141] introduces potential selection bias, as communities were chosen based on SWSW program engagement. This limits the representativeness of findings across broader populations. Can you include a sensitivity analysis or discuss how these biases might affect external validity? You may consider random sampling in future studies.
3. Provide additional descriptive statistics with measures of variability (e.g., Standard Deviation and Standard error) for key outcomes like time and distance. Also, consider inferential statistics to compare countries, as raw means/ranges alone limit interpretation. For instance, quantitative results (e.g., mean caloric expenditure of 231 cal [Line 41]) lack measures of variability (e.g., standard deviation) or statistical tests to compare across countries. Describe MEANs with standard errors or conduct ANOVA to test country-specific variations.
4. Include example quotes in the results to illustrate themes (e.g., mental burden). Also, you can use a framework like thematic analysis to structure findings. Also, while themes like risks from terrain and animals [Line 37] are noted, the absence of direct quotes or thematic coding limits the richness of experiential data. You can incorporate representative quotes and a thematic framework (e.g., grounded theory) to enhance qualitative rigour.
5. Discuss how dry-season data collection may overestimate burdens - data collection during the dry season in Zimbabwe [Line 186] and variable seasons across countries may skew results (e.g., longer distances in Kenya, 15.8 km [Line 40]), as water scarcity intensifies burdens [Line 80]. Can you analyze seasonal effects separately and leverage longitudinal data to assess variability?
6. Acknowledge the non-representative sample and suggest future population-based studies, if any.
7. Discuss how risks (e.g., accompanying women on journeys) were mitigated and how data privacy was ensured for the smart watch GPS.
8. Provide the DOI number for the listed references

For the Editor:

**Decision**

The paper may be accepted after the highlighted corrections are made.

**Professor Grace Oluwasanya**
