# Supplementary material for: Women’s experiences collecting and accessing water in Guatemala, Honduras, Kenya, and Zimbabwe: A mixed-methods investigation
Source: PLOS Glob Public Health. 2025 Dec 17;5(12):e0004355. doi: 10.1371/journal.pgph.0004355 (PMC12711095; doi:10.1371/journal.pgph.0004355)
Supplement: Attachment — Submitted filename: Caruso WJ Review Comment Responses PLoS GPH-2025 30OCT25.docx [file pgph.0004355.s010.docx]

To Whom in May Concern,

RE: " Women’s experiences collecting and accessing water in Guatemala, Honduras, Kenya, and Zimbabwe: A mixed-methods investigation " (PGPH-D-25-00317)

We are grateful to the reviewer for the time spent reviewing this manuscript.

Our point-by-point responses to all comments below, which proved helpful in improving the quality of this manuscript.

We have uploaded both ‘clean’ and ‘tracked changes’ versions of the manuscript to enable easier re-review by all. We hope these revisions are acceptable. Please do not hesitate to contact me with any questions.

We look forward to hearing from you.

On behalf of all authors,

Bethany Caruso

**Responses to Comments**

| **Journal Requirements** | | |
| --- | --- | --- |
| **Comments / requested revisions** | **Authors’ response** | **Additions /edits to manuscript**  **(as applicable)** |
| J1.  If the reviewer comments include a recommendation to cite specific previously published works, please review and evaluate these publications to determine whether they are relevant and should be cited. There is no requirement to cite these works unless the editor has indicated otherwise. | J1.  No recommendations made. | J1.  NA |
| J2  Please review your reference list to ensure that it is complete and correct. If you have cited papers that have been retracted, please include the rationale for doing so in the manuscript text, or remove these references and replace them with relevant current references. Any changes to the reference list should be mentioned in the rebuttal letter that accompanies your revised manuscript. If you need to cite a retracted article, indicate the article’s retracted status in the References list and also include a citation and full reference for the retraction notice. | J2.  We have reviewed the reference list.  No papers have been removed.  Two papers have been added that we were not aware of, but that were shared by the reviewer. | J2.  NA |

| **Reviewer 2 Comments** | | |
| --- | --- | --- |
| **Comments / requested revisions** | **Authors’ response** | **Additions /edits to manuscript**  **(as applicable)** |
| **Overall.**  This manuscript presents a mixed-methods study examining women's water collection experiences in four low-resource countries, combining qualitative insights (go-along interviews and observations) with quantitative measures (smart watches and scales) to quantify time, distance, energy, and other burdens. The research provides real-time evidence, addressing an important gap in understanding the gendered impacts of water insecurity, with implications for policy and interventions. The title is well-worded to capture the central theme of the paper. The abstract is concise and informative, the introduction provides a solid literature review, and the methods section details unique approaches using technology for precise measurements.  The strengths of the article include the multi-country scope, integration of mixed methods, and focus on underrepresented aspects like cognitive burden and energy expenditure – providing research-driven evidence to overlooked but important nuances. The collaboration with local partners also enhances contextual relevance. Other observations and corrections are highlighted below under respective subsections: | Thank you for your time reviewing. | **NA** |
| **Abstract**  (a) Line 41: "Mean caloric expenditure was 231cal (range: 36 (Guatemala) – 952 (Zimbabwe))." Units could be clarified for scientific accuracy. "Cal" is used, but in nutrition/energy contexts, it's typically "kcal" (kilocalories) to avoid confusion with small calories. Smartwatch data may likely estimate kcal. Kindly re-check.  (b) Line 30: Replace “experiences is” with “experiences are”  (c) Lines 38–42: The ranges (e.g., for time: 13 min in Guatemala to 287 min in Kenya) imply overall minimum-maximum values for specific countries. Clarify if these are extrema across all participants or per-country mins/maxes.  (d) Keywords: There are no keywords. Include about 4 – 6 keywords, arranged in alphabetical order | **Abstract**  (a) We appreciate the reviewer flagging this as the watches indeed measured kcal. We have changed ‘cal’ to ‘kcal’ throughout. In addition, we have added clarifying language in the methods.  (b) No change made. The verb ‘is’ is appropriate as the subject is investigation, which is singular. [The sentence reads as follows: “A current and comprehensive investigation of women’s water collection experiences is needed to understand their burden of this labor.”]  (c) An edit was made to clarify that these were the minimum and maximum times across the whole sample.  (d) Key words have been added. | **Abstract (*Underlined is new text)***  (a) **Change in methods:** ‘Watches were calibrated prior to the start of water collection using each participant’s weight, height, and age to estimate caloric expenditure; caloric expenditure is reported in kcal.”  (b) No change made to the text.  (c) “The mean water journey time (including going to the source, activities at the source, and returning) was 82 minutes (range across entire sample: 13 minutes (Guatemala) – 287 minutes (Kenya)).”  (d) Key words added: gender; energy expenditure; time use; unimproved water; unpaid labor; water fetching. |
| **Suggested Introduction copy edits**  (a) Line 57: Change ‘responsibility’ to ‘responsibilities’  (b) Line 67: Change ‘injury’ to ‘injuries’  (c) Line 85: Add ‘a’ before ‘…more precise’  (d) Line 94: Write ‘roundtrip’ to ‘round-trip’  (e) Line 102 – 103: Write ‘Traveled’ as ‘Travelled’ | **Suggested Introduction copy edits**  (a) change made  (b) Change not made. That would change what the cited paper reported. It did not say people reported injuries, but an injury.  (c) change made  (d) ‘Traveled’ is the correct spelling in American English. No changes made. | **Suggested Introduction copy edits (*Underlined is new text)***  (a) “These quantitative estimates of water collection responsibilities are vital for showing who bears this burden.”  (b) No edits made to text.  (c) “…reinforcing the need for a more precise understanding of the amount of time spent collecting water.”  (d)  No edits made to text. |
| **Suggested Methods copy edits**  (a) Line 134: Figure 1 is missing  (b) Line 150: Change ‘sample of individuals who were eligible’ to ‘sample of eligible individuals’  (c) Line 187: Change ‘was’ to ‘were’  (d) Line 208: Address missing Figure 1 | **Suggested Methods copy edits**  (a) Figure 1 is not missing. It is simply after Figure 2 in the PDF.  (b) We did not make this change as it changes the meaning of the sentence.  (c) No change. Data is plural.  (d) Figure 1 is not missing. It is after Figure 2 in the PDF. | **Suggested Methods copy edits**  (a) No edits made to text.  (b) No edits made to text.  (c) No edits made to text.  (d). No edits made to text. |
| **R3.1**  In Methods (Line 155; Page 8, Line 155): "In Guatemala, 95% of the population (91% rural; 98% urban) has at least basic drinking water service (i.e., from an improved source requiring no more than 30 minutes roundtrip for collection) (4)." You may revise as "In Guatemala, 95% of the population (91% rural; 98% urban) has at least basic drinking water service, defined as access to an improved source requiring no more than 30 minutes round-trip for collection per WHO/UNICEF JMP (4)." | **R3.1**  Suggested change made. | **R3.1**  ***Underlined is new text***  “In Guatemala, 95% of the population (91% rural; 98% urban) has at least basic drinking water service, defined as an improved source requiring no more than 30 minutes roundtrip for collection(4).” |
| **R3.2**  The use of convenience sampling [Line 150] and purposive community selection by World Vision [Line 141] introduces potential selection bias, as communities were chosen based on SWSW program engagement. This limits the representativeness of findings across broader populations. Can you include a sensitivity analysis or discuss how these biases might affect external validity? You may consider random sampling in future studies. | **R3.2**  We acknowledge the reviewer’s concern. Indeed, the very first limitation described in the limitations section is about the sample and how our sampling procedure limits external validity (generalizability).  We state the following: “Community sampling was purposive, and individual sampling was by convenience, thus data may not be representative, and are not generalizable.”  Unfortunately, it is not clear how a sensitivity analysis could be done with our current sample.  We will take the reviewer's suggestions for future studies into consideration | **R3.2**  No edits made to text. |
| **R3.3**  Provide additional descriptive statistics with measures of variability (e.g., Standard Deviation and Standard error) for key outcomes like time and distance. Also, consider inferential statistics to compare countries, as raw means/ranges alone limit interpretation. For instance, quantitative results (e.g., mean caloric expenditure of 231 cal [Line 41]) lack measures of variability (e.g., standard deviation) or statistical tests to compare across countries. Describe MEANs with standard errors or conduct ANOVA to test country-specific variations. | **R2.3**  We have included standard deviations; the reviewer may have missed these as they are next to the reported means (SD).  We have not conducted statistical testing to compare populations across countries, as these comparisons were not among our research objectives so would be outside the scope of this manuscript. | **R2.3**  No changes made |
| **R3.4**  Include example quotes in the results to illustrate themes (e.g., mental burden). Also, you can use a framework like thematic analysis to structure findings. Also, while themes like risks from terrain and animals [Line 37] are noted, the absence of direct quotes or thematic coding limits the richness of experiential data. You can incorporate representative quotes and a thematic framework (e.g., grounded theory) to enhance qualitative rigour. | **R3.4**  We appreciate that the addition of quotes can further enrich qualitative findings. For the purpose of this paper, we elected not to include quotes as we are covering many themes and we felt that that the quotes were not providing substantially more detail to the themes we were reporting on to warrant their inclusion here.  As we note in the methods, we did carry out thematic coding as part of our a rapid thematic analysis. Specifically, we note that: ‘The themes were chosen a priori based on the prompts in the data collection tools.”  We agree that grounded theory is a great analysis approach, however it is intended for generating a theory from the data. The purpose of this analysis was not to create a theory from the data, so we did not use that approach. We have added a statement to the limitations section indicating that further analysis of the qualitative data could be undertaken to answer additional questions. | **R3.4**  **(*Underlined is new text)***  “Finally, we carried out a rapid thematic analysis, which best suited our aim to understand women’s practices related to water collection. The qualitative data could be further explored in a subsequent analysis to understand determinants of those practices, if the data enables it.” |
| **R3.5**  Discuss how dry-season data collection may overestimate burdens - data collection during the dry season in Zimbabwe [Line 186] and variable seasons across countries may skew results (e.g., longer distances in Kenya, 15.8 km [Line 40]), as water scarcity intensifies burdens [Line 80]. Can you analyze seasonal effects separately and leverage longitudinal data to assess variability? | **R3.5**  This point is well taken. We have added a point in the limitations section about seasonal variability and the need to collect data during different seasons.  We cannot analyze seasonal effects as we collected data at only one time point, but have added that data collection at different time points is warranted. | **R3.5**  **(*Underlined is new text)***  “We only assessed a single journey to a water source at a single time of year per enrolled household due to time and resource constraints. Future research could collect data throughout the day and from all water collectors within a household to capture daily burden for individuals and the household more accurately. Further, data reflect the seasonal conditions during which data were collected. Previous research has shown that water fetching burden can be exacerbated during the dry season(24-26). Data were collected in Zimbabwe and Kenya during the dry season, and thus our data may reflect greater burden compared to what is experienced at other times of the year. Future research could assess variability of water collection and water work burden by collecting data during different seasons.” |
| **R3.6**  Acknowledge the non-representative sample and suggest future population-based studies, if any. | **R3.6**  As noted in response to R3.2, we note in the limitations that the sample may not be representative.  We state the following: “Community sampling was purposive, and individual sampling was by convenience, thus data may not be representative, and are not generalizable.”  While population-based studies have benefits, it would be very costly and hard to replicate the study we carried out at scale. As such, we do not make that recommendation explicitly. | **R3.6**  No edits made to text. |
| **R3.7**  (a) Discuss how risks (e.g., accompanying women on journeys) were mitigated and  (b) how data privacy was ensured for the smart watch GPS. | **R3.7**  (a) We have added a section on risk mitigation.  (b)  Data from smart watches were uploaded onto the Garmin Connect online platform. No names were attached to the watches; all watches had an ID that we assigned for tracking watches. Each ‘journey’ or observation was assigned a unique ID that did not include participant names. While participant information (weight, height, age) was used to calibrate the watch, it was updated for each new observation. Data were deleted from the watches after it was uploaded.  We have updated the methods with this information. | **R3.7**  (a) **New section on risk mitigation (underlined is new text):**  “Like all research, we acknowledge that this research could pose some unintentional risks. Specifically, we anticipated that the wearing of smart watches could cause conflict within families or communities or impose a safety risk to participants. We took steps to mitigate these risks by making it clear within communities that devices were not a gift but were to be returned to the research team. We clearly explained risks during consents so that women could opt out if they perceived that temporarily possessing a watch would create risk. Additionally, research can pose risks to those who collect data. To ensure the safety of our data collection team members, we hired and trained individuals from the broader research areas. We never had only one research team member accompany women on water journeys; water journeys were completed in teams of two. Finally, the research team intentionally worked with World Vision teams to ensure that research would not take place in areas with high perceived risks of banditry, where participants and research team members may be targets.”  (b) **New information in the methods (underlined is new text)**  “Data from the watches were synced to the ‘Garmin Connect’ web platform. No names were linked to the watches; each watch had an ID that the team used for tracking which watches were used for each journey (‘observation’). On the online platform, each observation was renamed with a unique participant ID; this ID did not contain any identifiable information. While some participant information (weight, height, age) was used to calibrate the watch in advance of the observed journey, this information was updated for each new journey observed and did not remain on the watch. Data were then downloaded and exported from the Garmin web platform as CSV and TCX files and uploaded onto a secure OneDrive folder. Data were deleted from the watches after it was uploaded. |
| **R3.8**  Provide the DOI number for the listed references | **R3.8**  This is not indicated to be a requirement of the journal, so we have not added the DOIs. We have formatted the references as instructed by the journal. | **R3.8**  No edits made to text. |
